# Restoration of floodplain meadows: Effects on the re-establishment of mosses

**DOI:** 10.1371/journal.pone.0187944

**Published:** 2017-12-11

**Authors:** Dorota Michalska-Hejduk, Grzegorz J. Wolski, Matthias Harnisch, Annette Otte, Anna Bomanowska, Tobias W. Donath

**Affiliations:** 1 Department of Geobotany and Plant Ecology, Faculty of Biology and Environmental Protection, University of Lodz, Lodz, Poland; 2 Department of Ecology and Environment, City of Riedstadt, Riedstadt, Germany; 3 Department of Landscape Ecology and Landscape Planning, Justus Liebig University Gießen, Gießen, Germany; 4 Department of Landscape Ecology, Institute for Natural Resource Conservation, Kiel University, Kiel, Germany; Indiana University Bloomington, UNITED STATES

## Abstract

Vascular plants serve as target species for the evaluation of restoration success as they account for most of the plant species diversity and vegetation cover. Although bryophytes contribute considerably to the species diversity of meadows, they are rarely addressed in restoration projects. This project is a first step toward making recommendations for including mosses in alluvial floodplain restoration projects. The opportunity to assess the diversity and ecological requirements of mosses on floodplain meadows presented itself within the framework of a vegetation monitoring that took place in 2014 on meadows located along the northern Upper Rhine. In this area, large-scale meadow restoration projects have taken place since 1997 in both the functional and fossil floodplains. Other studies have shown that bryophytes are generally present in green hay used in restoration, providing inadvertent bryophyte introduction. We compared bryophyte communities in donor and restored communities and correlated these communities with environmental variables—taking into account that the mosses on the restoration sites possibly developed from green hay. This analysis provided insights as to which species of bryophytes should be included in future restoration projects, what diaspores should be used, and how they should be transferred. Data on bryophyte occurrence were gathered from old meadows, and from restoration sites. We found distinct differences in bryophyte composition (based on frequency) in restored communities in functional flood plains compared to donor communities. Generally, restoration sites are still characterized by a lower species-richness, with a significantly lower occurrence of rare and red listed species and a lower species-heterogeneity. In conclusion, our research establishes what mosses predominate in donor and restored alluvial meadows along the northern Upper River, and what microsite conditions favour particular species. This points the way to deliberate introduction of moss diaspores for more complete alluvial meadow restoration.

## Introduction

In Europe, low productive wet meadows have a high conservation value because they are inhabited by many valuable and protected plant and animal species [1–7]. Consequently, species-rich floodplain meadows are protected by the Fauna Flora Habitat Directive of the European Union (Council of the European Union 1992). Today, the biggest threats to these ecosystems are either the intensification or the abandonment of agricultural utilisation, the latter of which results in secondary succession [8–11]. Therefore, these unique species-rich habitats are a focus of conservation and restoration activities [7, 12–17].

Projects associated with the restoration of semi-natural non-forest communities are mainly connected to fen meadows [18–22] and calcareous grasslands [14, 23–26], but are also linked to alluvial meadows [27–33]. A common finding of all these projects was that—as long as biotic and abiotic prerequisites are met—the active restoration of species-rich grasslands is an effective means for the re-establishment of rare and endangered vascular plant species and communities [10, 15–16]. For example, 101 vascular plant species were successfully re-established on alluvial meadows along the northern Upper Rhine only three years after restoration measures took place, including 28 species of the Red List [32].

Vascular plants have largely been given priority in wetlands restoration [19, 23, 34–36], even in ecosystems where mosses are a crucial component of the vegetation [37–39]. Including bryophytes in wetland restoration projects will increase species richness and vertical diversity, and help to create a vegetation structure closer to old and communities with pre-industrial disturbance regimes [40–41]. Moreover, mosses play an important role in water balance, the nutrient cycle and in the creation and modification of habitats occupied by other organisms [42]. They form a vegetation layer beneath the vascular plants which results in many mutual and unidirectional interactions between bryophytes and vascular plants [25, 43–47]. Since bryophytes play an important role in the species composition and function of fen systems [21–22, 48–49] they are the focus of fen restoration research [21, 38–41, 46, 49, 50–51]. However, this group of plants is not addressed in floodplain meadow restoration projects. Despite this, several studies have proved that bryophytes can be successfully transferred with hay and raked material [16, 26, 52–54], and therefore mosses should also be taken into account in the planning and execution of measures aiming at the re-establishment of such semi-natural communities [26].

The restoration technique, used and tested largely in the projects along the northern Upper Rhine, was the transfer of fresh green hay, i.e. freshly cut plant material [16] from donor sites with high species richness into restoration fields of similar ecological conditions [29–30, 32–33]. However, it was only in 2014—after nearly 20 years of restoration practice—that we also turned our attention to mosses, and decided to assess how they react to restoration measures. Therefore our research was carried out in response to the following questions: i) are there differences with regard to the species richness and diversity of bryophytes on the donor and restoration sites?; ii) are there species typical for restoration and donor sites, do they differ in their ecological characteristics?; iii) does the localization of restoration sites either in the fossil floodplain or the functional floodplain have any influence on the bryophyte flora?

## Materials and methods

### Study area

The study was conducted on floodplain meadows in the Holocene flood plain of the northern Upper Rhine, approx. 30 km south-west of Frankfurt am Main, Germany (49°55'N 8°22'E– 49°48'N 8°27'E). The region is one of the last and most important strongholds of many rare and endangered alluvial grassland species in Europe [55]. A winter dyke divides the area into two hydrological compartments: the functional and the fossil floodplain [29]. Although the winter dyke prevents direct flooding of the fossil floodplain by river water, depressions are regularly submerged by clear seepage groundwater. In contrast, the functional floodplain riverwards of the dyke is directly exposed to flooding by river water up to 3m above the terrain [56]. In consequence the ground-water table shows strong seasonal and inter-annual fluctuations [56]. A combination of clay rich-soils (>60%; [57]), the relatively warm and dry climatic conditions with a mean temperature of 10.3°C, and a mean annual precipitation of 580 mm [58] lead to large variations in available plant soil moisture especially during the summer. The soils are calcic vertisols, which have developed from the latest Holocene aggradation of the Rhine and consist of calcic, clay-rich material containing many mollusk shells [57].

Until the 1950s, the area was dominated by species-rich alluvial grasslands that were managed extensively as hay meadows [59]. Due to intensified drainage and structural changes in agriculture, many sites were subsequently converted into arable fields [60]. In the 1980s, however, re-conversion into grassland began after severe flooding events had rendered arable farming uneconomical, and as a consequence, the land was set aside for conservation purposes. As restoration by natural succession met with little success in the case of floodplain meadows due to a strong dispersal limitation of the target species [61–62], several large-scale restoration projects were initiated by the Department of Landscape Ecology and Landscape Planning, University of Giessen (Hesse, Germany; [29–30, 32]). At the restoration sites of the current study, typical floodplain meadows (ca 57 ha) were re-established by means of green hay transfer from donor sites (see [29–30, 33] for details). Donor sites are meadows that are characterised by an exceptionally high proportion of typical, rare and endangered floodplain meadow species [29–30] and cover about 14.28 ha. These meadows were continuously used as hay meadows for decades. Most of the donor sites are only mown once a year starting from mid- September until the end of October. Only some undergo two cuts a year, with the first cut around the middle of June and the second—which is used for green hay transfer—usually in September/October [30, 33].

### Sampling design and data collection

Bryophyte data were collected in May and June 2014 both on donor and restoration sites. The survey comprised a total of 207 plots of 10 x 10 m from 24 sites located along both sides of the main dyke of the Rhine. 60 plots are located on donor sites, of these 40 lie in the fossil and 20 in the functional floodplain. 147 plots are on restoration sites—66 in the fossil floodplain and 81 in the functional floodplain. In each plot, five samples of mosses were collected along both diagonals of the plot. Additionally, total cover of the mosses and the herb layer was estimated on the plots located on the restoration sites. The sampling was done by Matthias Harnisch (donor sites) and Dorota Michalska-Hejduk (restoration sites). All donor sites, from which mosses were collected, are in public ownership of the Federal State of Hesse, which is one partner of the floodplain meadows restoration projects. The permission to collect the samples was given by the owner. All restoration sites assessed, thus, are all in the ownership of the City of Riedstadt which is a major partner of the restoration projects, so free access and sampling was granted here, too.

The cover and abundance values of vascular plant species were evaluated using a modified Braun–Blanquet-scale [63]. Quantitative data derived from the relevés were converted into an average percentage cover according to the following rule: r = 0.1; + = 1; 1 = 2.5; 2m = 4.25; 2a = 7.5; 2b = 20; 3 = 37.5; 4 = 62.5; 5 = 87.5. In the current study this data was used to derive weighted Ellenberg ecological indicator values (R—soil reaction, M—moisture value, N—nutrient value; [64]), and the Shannon diversity and evenness indices.

The occurrence frequency of particular bryophyte species is described in relation to their distribution in the whole study area, therefore applying the following levels of frequency: very rare (1 to 17 findings), rare (18–35), frequently (36−53), numerous (54−71) and common (72−90 findings).

The categories used to describe the general ecological requirements of species and their socio-ecological classification were taken from Dierßen [65], generalizing his classes as follows:

**Humidity:** Hyd (hydrophyt—adopted to tolerate inundation), Hig (e hygrophyt—extremely wet, h hygrophyt—very wet, c hygrophyt—considerably wet), Mes (mesophyt—moderately wet) and Xer (c xerophyt—moderately dry, h xerophyt—very dry). The remaining classes (Hyd-Hig, Hig-Mes, Hig-Xer, Mes-Xer) contain species with a wide range in regard to humidity, which are put by Dierßen [65] into two or more groups.

**Light:** Scio (h sciophyt—adopted to minimum light supply, c sciophyt—considerably adopted to shade), Phot (m photophyt—in moderately illuminated habitats, c photophyt—in moderately or considerably lighted sites, h photophyt—growing in full light). The combined class Scio-Phot consists of species with a large ecological amplitude, growing in both shade and full light.

**Substrate reaction:** Aci (e acidophyt: ph < 3.3 − extremely acidic, h acidophyt: ph 3.4−4.0 − highly acidic, c acidophyt: ph 4.1−4.8 − considerably acidic, m acidophyt: ph 4.9−5.6 − moderately acidic), Sub (subneutrophyt: ph 5.7−7.0 –subneutral), Basi (basiphyt: ph > 7 –basic). The remaining classes (Aci-Basi, Aci-Sub, Sub-Basi and Eury) comprise species tolerant of a wide range of pH-value, which are listed in two or more categories by Dierßen [65].

The nomenclature of the taxa follows Hill et al. [66]. The red list categories follow Ludwig et al. [67], Drehwald [68] and LUBW [69].

### Data analysis

To analyze the differences in the composition of the bryophyte communities at the donor and restoration sites as well as at the restoration sites situated in the fossil or functional flood plain we employed Non-metric multidimensional scaling ordination (NMDS; [70]). The ordination was conducted using the Sørensen-distance measure. A secondary matrix was used to overlay the ordination diagram with vectors of vegetation derived site parameters, such as Ellenberg indicator values, open soil or diversity measures.

Significant indicator species for restoration or donor sites as well as for restored sites situated in the fossil or functional flood plain were identified through an Indicator Species Analysis [71]. The obtained values were tested for significance with a Monte-Carlo permutation test.

T-tests were applied to check significant differences in the total number of bryophyte species and differences in the number of red listed species between donor and restoration sites across both floodplain compartments as well as to test differences between the functional and the fossil floodplains.

The statistical analyses were performed with the STATISTICA 13 software package [72]. The Non-metric multidimensional scaling (NMDS) ordination and the Indicator Species Analysis were conducted using PC-Ord 5.19 [73].

## Results

### Species richness of the bryophyte flora

In the course of the vegetation survey, 27 species of mosses were found, belonging to 23 genera (S1 Table). Among these, the genera *Brachythecium* (with four species) and *Plagiomnium* (two species) showed the broadest distribution, whereas all other genera were represented by only one species. The most predominant species were *Calliergonella cuspidata* (Hedw.) Loeske (90 findings), *Oxyrrhynchium hians* (Hedw.) Loeske (57) and *Brachythecium rutabulum* (Hedw.) Schimp. (54). In contrast, most species were found only once (S1 Table). The plagiotropic mosses form the largest morphological group with 19 species found with considerably less belonging to the orthotropic group (8). The highest frequency of occurrence among plagiotrophic species *Calliergonella cuspidata*, *Pseudoscleropodium purum* (Hedw.) M.Fleisch. and *Drepanocladus aduncus* (Hedw.) Warnst, whereas among the orthotropic mosses e.g.: *Fissidens adianthoides* Hedw. were found most frequently.

### Floristic composition of donor and restoration sites

In comparison to the restoration sites the species richness of the donor sites is almost twice as high. On the donor sites 24 species were found, 13 of which are exclusively connected to these meadows (Table 1 and S1 Table). Among these, the most frequent were *Campyliadelphus chrysophyllum* (Brid.) R.S.Chopra (11 findings), *Thuidium delicatulum* (Hedw.) Schimp. (9), *Campylium stellatum* (Hedw.) Lange & C.E.O.Jensen (4) and *Plagiomnium undulatum* (Hedw.) T.J.Kop. (with 4 findings)—for these species the differences between donor and restoration sites are statistically significant (S1 Table). On the restoration sites only 14 species were found, three of which did not occur on the donor sites. Thus, 11 species grew on both donor and restoration sites of which *Calliergonella cuspidata*, *Oxyrrhynchium hians* and *Brachythecium rutabulum* were the most frequent. The rarest among the recorded species on both sites were *Barbula convoluta* Hedw. and *Sciuro-hypnum oedipodium* (Mitt.) Ignatov & Huttunen (S1 Table). The donor sites differed from the restoration sites in having a significantly higher number of red listed bryophyte species as well (Table 1).

**Table 1:** Differences in total species number of bryophytes and number of red listed bryophyte species [67–68] between donor and restoration sites as well as between functional and fossil floodplain.

	**donor sites**		**restoration sites**			
**(df = 215)**	**mean**	**±SE**	**mean**	**±SE**	**t-value**	***P***
# species	2.69	±0.17	1.29	±0.09	8.10	**<0.0001**
# red list species	0.67	±0.10	0.22	±0.04	5.08	**<0.0001**
**Flood plain compartment**	**functional**		**fossil**			
**donor & restoration sites (df = 215)**	**mean**	**±SE**	**mean**	**±SE**	**t-value**	***P***
# species	1.38	±0.13	2.06	±0.12	3.84	**0.0002**
# red list species	0.21	±0.04	0.51	±0.07	3.54	**0.0005**
**donor sites (df = 68)**						
# species	2.10	±0.27	2.92	±0.20	2.26	**0.027**
# red list species	0.30	±0.11	0.82	±0.13	2.35	**0.022**
**restoration sites (df = 145)**						
# species	1.20	±0.14	1.41	±0.10	1.20	0.23
# red list species	0.19	±0.05	0.27	±0.06	1.22	0.23

Df—degrees of freedom; SE—standard error; *P*-values < 0.05 are in bold.

Comparing the occurrence of bryophytes on donor sites with restoration sites, the largest statistically relevant differences in frequency can be observed in regard to three species: *Calliergonella cuspidata*, *Pseudoscleropodium purum* and *Plagiomnium affine* (S1 Table).

Looking at the growth form, plagiotropic mosses prevail on both types of meadows, with 16 species (67%) on donor sites and 11 (79%) on restoration sites. Thus, orthotropic bryophytes numbered considerably fewer with only eight species (33%) on donor sites and three species (21%) on restoration sites. However, taking the frequency of species into account these differences are not significant.

**Fig 1:**
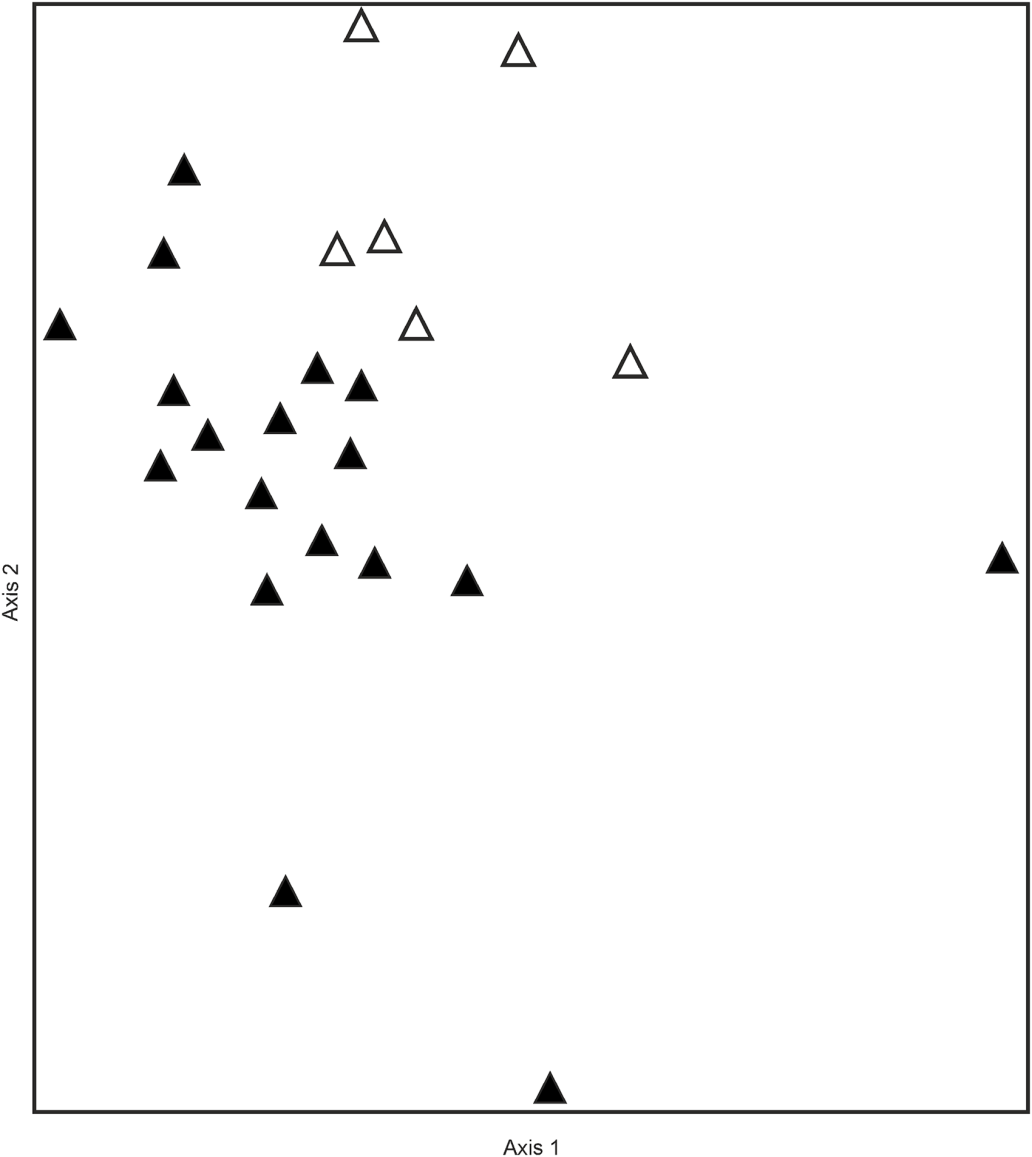
NMDS Ordination of bryophyte relevés of donor sites (open symbols) and restoration sites (filled symbols). Please note that for this analysis we merged data at site level. The final stress of the 2-dimensional solution was 13.7.

In addition, the NMDS ordination revealed clear differences in the species composition of donor and restoration sites (Fig 1). While donor sites are widely spread, indicating a higher heterogeneity in the bryophyte composition, restoration sites seem to be more homogeneous with regard to species occurrence. Therefore, the latter are grouped in the upper right half of the ordination space.

### Ecological requirements of bryophytes

**Fig 2:**
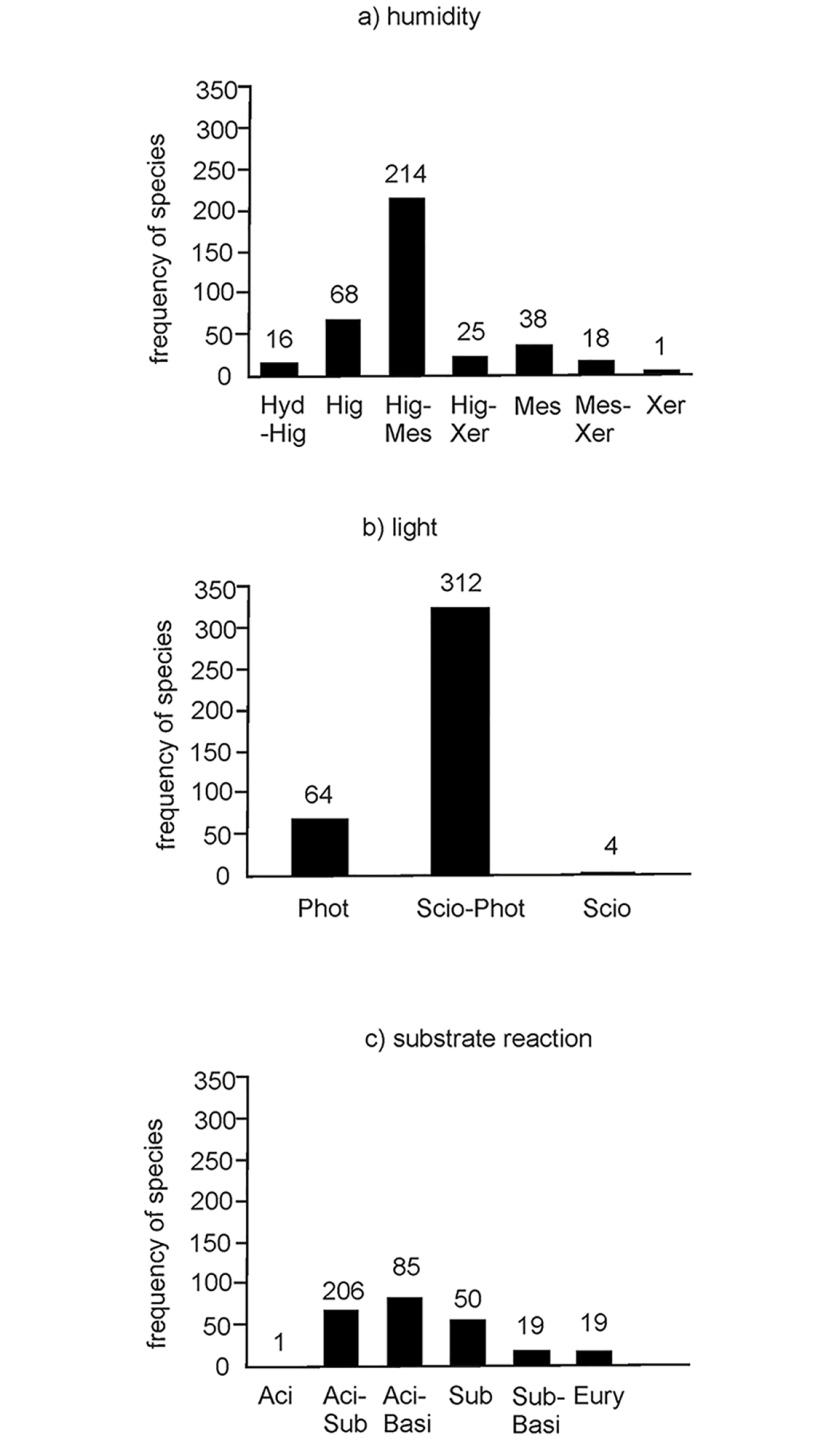
General ecological requirements of the bryophyte species found in the researched meadows. (a) Humidity: Hyd—hydrophyte, Hig—hygrophyte, Mes—mesophyt, Xer—xerophyte, Hyd-Hig, Hig-Mes, Hig-Xer, Mes-Xer—contain species with a wide range with regard to humidity, put into two or more groups by Dierßen (2001). (b) Light: Scio—sciophyt, Phot—photophyt, Scio-Phot—large ecological amplitude, growing in both shade and full light. (c) Substrate reaction: Aci—acidophyt, Sub—subneutrophyt, Basi—basiphyt), Aci-Basi, Aci-Sub, Sub-Basi, Eury—species tolerant of a wide range of pH-value, which are listed in two or more categories by Dierßen [65].

**Fig 3:**
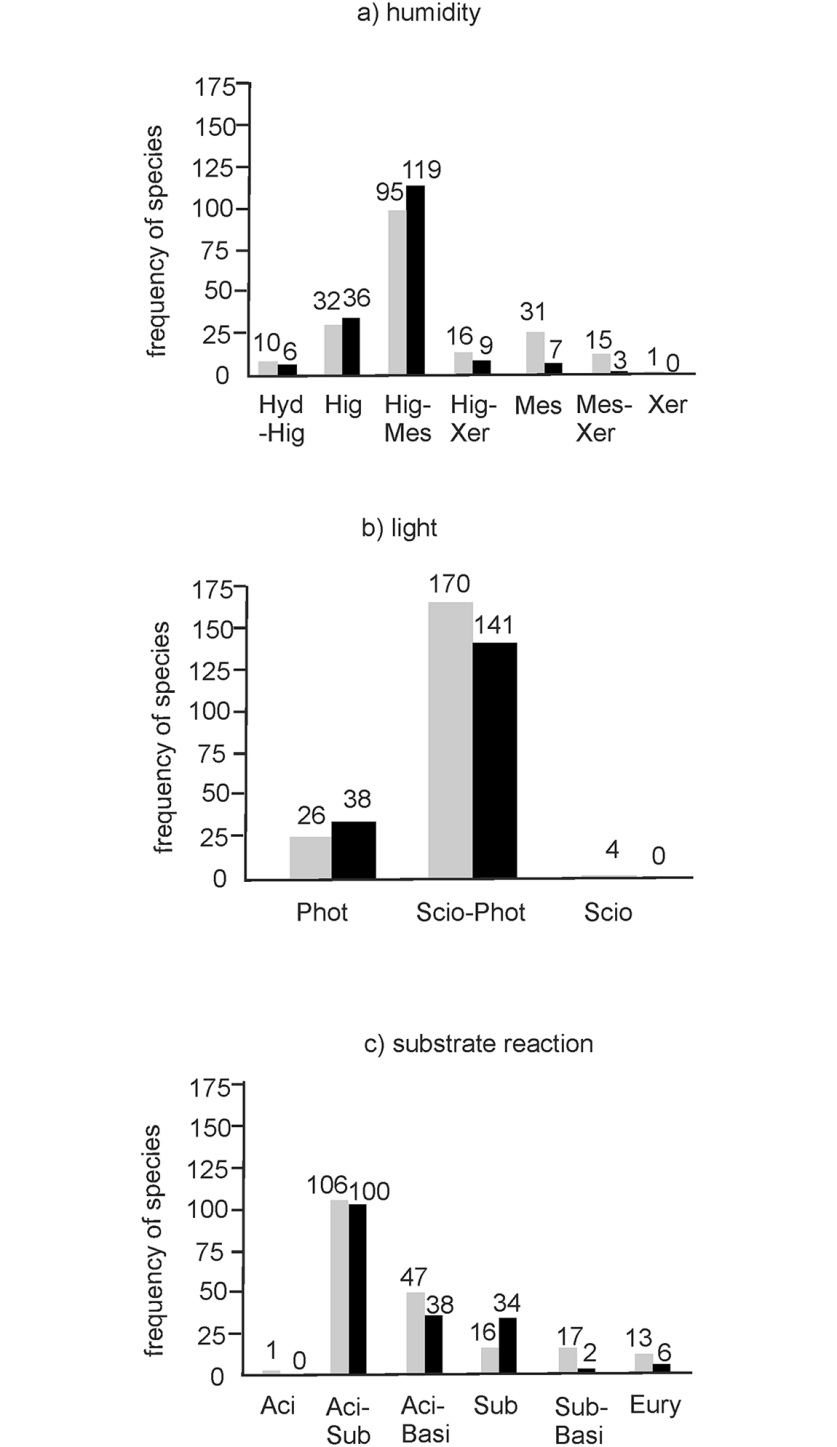
Ecological requirements of the bryophyte species found in the donor (gray bars) and restoration (black bars) meadows. (a) Humidity. (b) Light. (c) Substrate reaction. Explanations: see Fig 2.

In regard to humidity, species tolerating moderately to extremely wet sites (Hig-Mes) were predominant with 214 recordings. In this group the species *Calliergonella cuspidata* (90 recordings), *Oxyrrhynchium hians* (57) and *Brachythecium rutabulum* (54) were the most frequent. Noticeably fewer occurrences were recorded for groups connected exclusively with wet habitats (Hig, 68) and moderately wet habitats (Mes, 38). Interestingly, *Drepanocladus aduncus* was the only species found that temporarily tolerates inundation (16 recordings; Fig 2). On restoration sites species that prefer wetter habitats (extremely to moderately wet, Hig and Hig-Mes) prevail, whereas on donor sites more species belong to the mesophytic and xerophytic groups. The only exception is *Drepanocladus aduncus*–a species that temporarily tolerates inundation (Hyd)–which was to be more frequently found on donor sites than on restoration sites (Fig 3).

As for light, ubiquitous species with a wide range from those considerably adapted to shade to those that grow in full light (group Scio-Phot) were predominant on both donor and restoration sites. While only one species, *Plagiomnium undulatum*, was found that is adapted to minimum light supply and characteristic for forest communities (sciophyt-group; Scio), seven species growing only in moderate and full light (photophytes, Phot) were recorded (Fig 2). Species tolerating shade (Scio) were registered only on donor sites, but on restoration sites species growing only in full light occurred more frequently (Fig 3).

The majority of mosses found belong to the group tolerating a pH-value between 4.1 and 7 (Aci-Sub, 14 species with together 206 recordings). Only two species—*Oxyrrhynchium hians* (57 recordings) and *Pseudoscleropodium purum* (28)–tolerate a higher pH-value (from pH 4.1 to > 7). Only one species, *Warnstorfia fluitans*, (Hedw.) Loeske is stenoecious and prefers acidic soils with a pH-value of between 4.1 and 4.8 (Fig 2). The composition of mosses on the donor sites appears to be more balanced. Only one species (*Warnstorfia fluitans*), which belongs to the group of acidophilic bryophytes and needs a pH-value of 4.1 to 4.8, was found exclusively on a donor site. In addition, species which are more alkaliphilic (Sub-Basi) with a pH-range of 5.7 to 7 were more often recorded on donor sites (Fig 3).

### The impact of restoration site localization on bryophyte flora

The restoration sites in both the functional floodplain and fossil floodplain show a similar number of bryophyte species as well as roughly similar frequencies In restoration fields in the fossil floodplain, 12 species were found (94 recordings) whereas 11 species were located in the functional floodplain (86 recordings).

**Fig 4:**
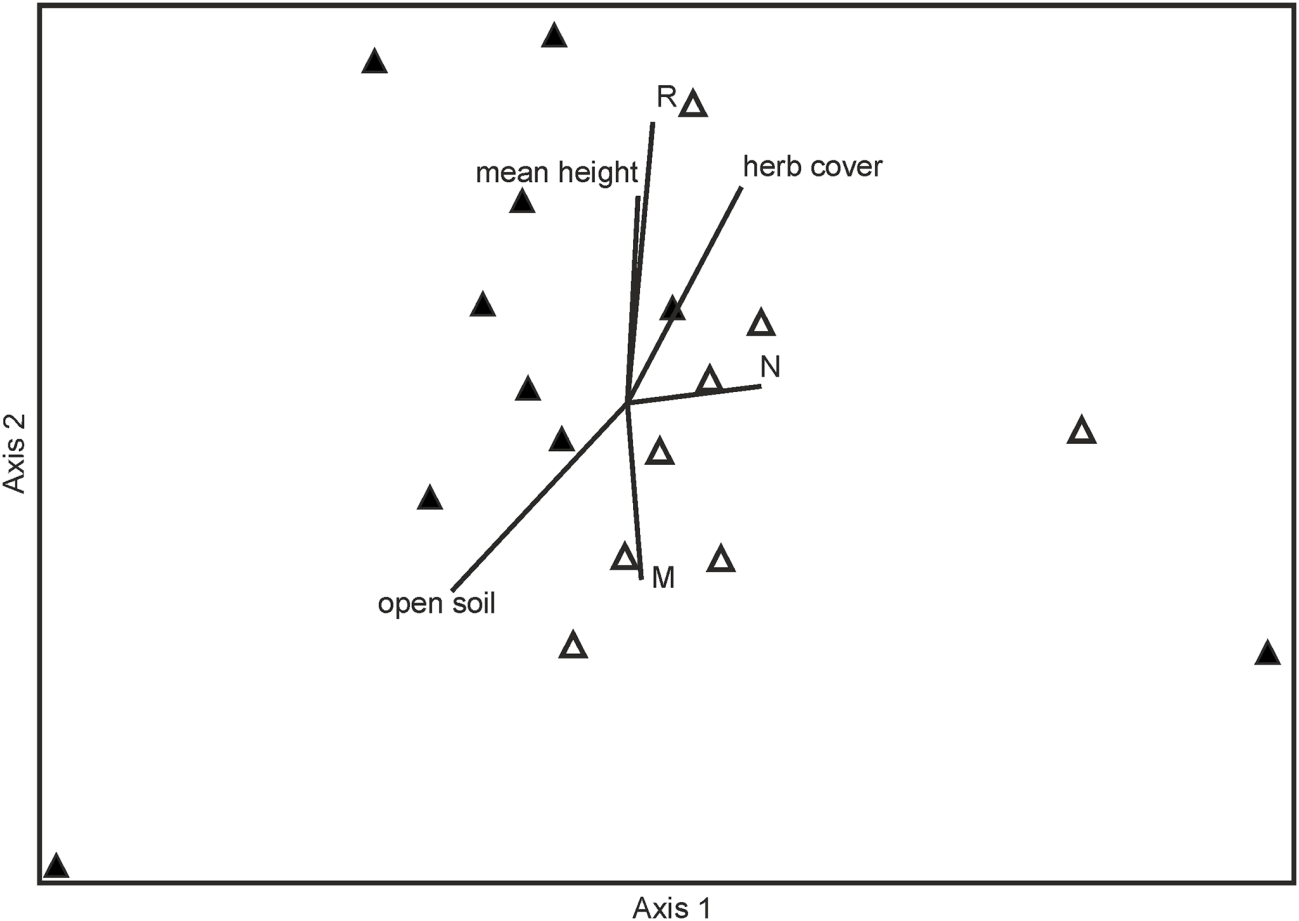
NMDS Ordination of bryophyte relevés of restoration sites situated in the fossil floodplain (open symbols), functional (filled symbols) flood plain, and vectors for vegetation derived site variables (r_axes_ > 0.4). Please note that for this analysis we merged data at site level. The final stress of the 2-dimensional solution was 10.2. R = Ellenberg soil reaction value; M = Ellenberg moisture value; N = Ellenberg nutrient value.

However, in spite of these similarities they differ with regard to community composition as defined by species frequency. On the restoration sites in the fossil floodplain the three species *Brachythecium mildeanum* (Schimp.) Schimp. (15 recordings), *Calliergonella cuspidata* (24) and *Brachythecium rutabulum* (32) were the most frequent. Furthermore, three species were found only in the fossil floodplain, namely *Brachythecium albicans* (Hedw.) Schimp, *Brachythecium rivulare* Schimp. *and Sciuro-hypnum oedipodium*. These differences also appear in the NMDS-ordination in a clear distinction between reléves situated in the fossil and those situated in the functional floodplain (Fig 4). Axis 1 represents a gradient of increasing nutrient availability (N, r = 0.47) indicating higher nutrient availability in the fossil than in the functional flood plain. In contrast, Axis 2 represents a negative gradient of Ellenberg moisture value (r = −0.53) and an increasing Ellenberg reaction value (r = 0.68). At the same time the vegetation parameters, the “number of vascular plant species”, “mean height” and “cover of the vascular plant species” increases along axis 2 (r = 0.40, r = 0.59 and r = 0.60, respectively). In addition, herb cover increases along Axis 1 and 2 (r = 0.43 and r = 0.60, respectively), while the proportion of open soil decreases (r = −0.54 and r = −0.56, respectively) along both Axes.

There was only one significant indicator species for the fossil floodplain, *Brachythecium rutabulum* (indicator value = 36.3; p = 0.0076) and none for the functional floodplain (S1 Table).

However, on the restoration sites in the functional floodplain *Brachythecium mildeanum* (14 recordings), *Calliergonella cuspidata* (16) and *Oxyrrhynchium hians* (27) were the species most frequently observed. In addition, the mosses *Hygroamblystegium varium* (Hedw.) Mönk and *Plagiomnium affine* could only be found in the functional floodplain.

Analyzing the ecological conditions of the restoration sites in both the functional and fossil floodplains, no statistically significant differences were discovered, although the mosses detected microsite differences, as previously discussed. With regard to light, the meadows in both compartments had nearly identical characteristics, whereas humidity and soil reaction only differed in single recordings.

However, on the sites in the fossil floodplain a greater number of Red List species were found as was the case with the restoration fields (Table 1).

## Discussion

As the obtained floristic data from restoring floodplain meadows show, the species composition of mosses differ between restoration sites and ancient meadows (donor sites). This may be an effect of green hay composition and the colonization potential of the mosses species that are transferred. It confirms the conclusions by Jeschke [26] on calcareous grasslands. Of course, it should be remembered that the mosses species investigated in the northern Upper Rhine case study were only transferred with green hay inadvertently because the project's focus was on vascular plant restoration.

The assessment of the obtained data showed that the most important environmental factors affecting the occurrence of bryophytes on restoration plots are light and humidity. According to the results obtained by Rochefort et al. [41] and Graf and Rochefort [38] the same factors have the greatest impact on the regeneration of fen mosses. Experiments conducted in the field as well as in greenhouses show that fen bryophytes exhibit a significantly higher regeneration rate under dense shade as for example under large herbaceous plants such as *Scirpus sp*. [38]. Nevertheless, this ability of the moss species to develop better in shaded environments is not only a result of photoinhibition [74] but is initiated by the humidity of substrate and a moderate microclimate [75]. Similar reactions of bryophytes were observed in calcareous grasslands where the water holding capacity of herbaceous litter allowed for a higher growth of bryophytes [76]. Graf & Rochefort [38–39] suggest that air humidity is more important to moss growth than substrate humidity. Our research shows that in restoration meadows the bryophyte cover is not linked to the density of vascular plants. Only five (*Drepanocladus aduncus*, *Brachythecium mildeanum*, *Barbula convoluta*, *Calliergonella cuspidata* and *Amblystegium serpens* (Hedw.) Schimp. of the analysed species are considered as preferring shade [65]. On the other hand, we could not verify a higher lushness of mosses on habitats with a less light. Furthermore, with regard to light no differences between the species on donor sites and restoration sites could be found. These results imply that the variable “light” had no influence on the introduction of bryophyte species on newly created meadows. The mosses reaction to the “soil humidity” factor was therefore slightly different: in comparison with the donor sites, where species preferring moderately dry to dry soils were predominant, on the restoration sites species tolerating wetter habitats, from moderately to extremely wet were more prevalent.

One of the most important variables in connection to the “water” factor having an influence on the dispersal of mosses in restored plant communities is the flooding of habitats. Lengthy inundation limits the growth of bryophytes, mainly due to physical disturbance such as erosion and sedimentation [77]. Therefore, fen restoration sites, where the water table is just below the surface, should show the highest rate of moss regeneration [38]. In our case, a similar number as well as frequency of species characterize the restoration sites, regardless of their location in the functional or fossil floodplains. They do not vary in regard to the environmental variables, whereas the composition of species as well as the prevalent species are in fact different. An important observation seems to be that rare species of the bryophyte Red List were found in meadows on the fossil floodplain, which is flooded only by ascending ground water and not by the flooding of the Rhine. This provides evidence supporting the hypothesis of Quinty and Rochefort [77] concerning the inhibition of mosses' growth on more frequently flooded sites. At the same time, the habitats in the fossil flood plain, showed a higher variety of bryophyte species in regard to their humidity requirements. For example, both *Brachythecium rivulare*, and *Brachythecium albicans*, which prefer wet riverside and very dry sites, respectively, occur in the fossil floodplain. This provides evidence of a higher variability of water conditions on the fossil floodplain.

Our results showed that bryophytes inadvertently introduced in "green hay" (or arriving from other sources) sorted themselves out, surviving where microsite conditions matched the ecological requirements of each species. Similar species sorting has been described in numerous projects concerning moss regeneration in bog restoration [38–39, 41, 50–51, 77–78].

It is important to find a suitable way for the transfer of diaspore material [39]. In restoring degraded peat land, one of the proposed methods for the reintroduction of bryophyte species, lost due to drainage, is to use gametophyte fragments as propagules [39, 41, 50–51, 78]. Because this method has been tested on mosses with a high capacity for regeneration (mainly *Sphagnum* and *Polytrichum* species), it seems feasible for mosses characteristic of smaller species-rich fens [41, 50, 78]. However, as the success of the diaspore reintroduction technique was not confirmed in a large-scale restoration project, this measure is rather not applicable for the restoration of large grassland habitats as described in this paper where restored meadows cover over 57ha.

Another tested method is the transfer of cryptogam species with green hay and raked material [16, 26, 52–54, 79]. According to Jeschke [26], green hay transfer is a reasonable method of transferring the majority of vascular plant species, pleurocarpous mosses and also liverworts onto restoration sites. So this should be a suitable measure to establish new meadows with high mosses species richness. The author suggests that combining different methods of restoration is the most effective for the successful restoration of non-forest communities, e.g. green hay transfer in patches in combination with raked material. This combined method is adequate for the transfer of acrocarpous moss and epigaeic lichen species as well as some xerothermic vascular plants. However, our results show a higher heterogeneity of the bryophyte composition on the donor sites while the restoration sites seem to be more homogeneous in regard to species occurrence. Furthermore, the donor sites are distinguished from the restoration sites by a significantly higher number of red listed bryophyte species. While the differences in the bryophyte community might be an indication that the transfer of green hay alone is not sufficient as a method for the introduction of mosses and especially rarer bryophyte species on restoration sites, the lower heterogeneity might also be a result of the time period the bryophyte community has had to develop in restored versus donor sites on fossil floodplains. Schmalholz and Hylander [80] found that bryophyte communities need at least 40 years to fully develop. Interestingly, there seems to be no link between the cover of bryophyte species and the cover of vascular plants in our study, which is in contrast to the findings of other open field studies [81].

Our findings are a promising beginning for developing strategies to use bryophytes in floodplain meadow restoration projects. As shown in this study green hay could be deliberately selected to include bryophytes that thrive in restored meadows.

## Conclusions for restoration management

Our findings show that in order to restore an adequate diversity of species in a habitat it is necessary not only to pay attention to vascular plants but also to apply additional methods for mosses to be re-established. As the bryophyte flora of floodplain meadows differs significantly between the donor and restoration sites in terms of species richness and diversity, mosses should be taken into account in the restoration and conservation measures in floodplain meadows as well.

Even though green hay transfer enables the restoration of habitats to be nearly as species rich as donor sites are in vascular plants, it does not guarantee an equivalent richness in bryophyte species. It is possible to restore floodplain meadows that are both rich in vascular plants and mosses species, but this will require a combination of methods. No single restoration method can be universally recommended, as the method must be adapted to the species composition of the target site and to edaphic factors.

## Supporting information

S1 Table Frequency and treatment of bryophytes occurring on investigated meadows. P-values < 0.05 are in grey. IV—Indicator Value, TO—total, DO—donor sites, RE—restoration sites, FO—fossil flood plain sites, FU—functional flood plain sites, RLH—red list of mosses in Hessia [68], DY—dynamic of populations of threatened mosses in Hessia [68], RLG—red list of mosses in Germany [67], 2 –critically endangered, 3 –endangered, V—near threatened, * not threatened, D—data deficient;? –no data, ↓ –slight declining trend, = stabile population.
(DOCX)
