# Supplementary material for: Restoration of floodplain meadows: Effects on the re-establishment of mosses
Source: PLoS One. 2017 Dec 11;12(12):e0187944. doi: 10.1371/journal.pone.0187944 (PMC5725158; doi:10.1371/journal.pone.0187944)
Supplement: S1 Table — P-values < 0.05 are in grey. IV—Indicator Value, TO—total, DO—donor sites, RE—restoration sites, FO—fossil flood plain sites, FU—functional flood plain sites, RLH—red list of mosses in Hessia [68], DY—dynamic of populations of threatened mosses in Hessia [68], RLG—red list of mosses in Germany [67], 2 –critically endangered, 3 –endangered, V—near threatened, * not threatened, D—data deficient;? –no data, ↓ –slight declining trend, = stabile population. (DOCX) [file pone.0187944.s001.docx]

|  | Frequency | | | IV | p * | Frequency | | IV | p * | RE | | IV | p * | RLH | DY | RLG |
| --- | --- | --- | --- | --- | --- | --- | --- | --- | --- | --- | --- | --- | --- | --- | --- | --- |
|  | TO | DO | RE |  |  | FO | FU |  |  | FO | FU |  |  |  |  |  |
| **Species occur only on donor sites** |  | | | | | | | | | | | | | | | |
| *Abietinella abietina* (Hedw.) M.Fleisch. | 1 | 1 | 0 |  |  | 0 | 1 |  |  | 0 | 0 |  |  | V | ↓ | V |
| *Bryum pseudotriquetrum* (Hedw.) P.Gaertn. et al. | 1 | 1 | 0 |  |  | 1 | 0 |  |  | 0 | 0 |  |  | 3 | ↓ | V |
| *Campyliadelphus chrysophyllum* (Brid.) R.S.Chopra | 11 | 11 | 0 | **33.3** | **0.0596** | 11 | 0 | **7.9** | **0.0072** | 0 | 0 |  |  | V | ? | V |
| *Campylium stellatum* (Hedw.) Lange & C.E.O.Jensen | 4 | 4 | 0 | **33.3** | **0.0596** | 2 | 0 |  |  | 0 | 0 |  |  | 2 | ↓ | 3 |
| *Cirriphyllum piliferum* (Hedw.) Grout | 1 | 1 | 0 |  |  | 0 | 1 |  |  | 0 | 0 |  |  | * | = | * |
| *Cleistocarpidium palustre* (Bruch & Schrimp.) Ochyra & Bednarek-Ochyra | 2 | 2 | 0 |  |  | 2 | 0 |  |  | 0 | 0 |  |  | 3 | ↓ | 3 |
| *Eurhynchiastrum pulchellum* (Hedw.) Ignatov & Huttunen | 2 | 2 | 0 |  |  | 2 | 0 |  |  | 0 | 0 |  |  | 2 | ? | D |
| *Funaria hygrometrica* Hedw. | 1 | 1 | 0 |  |  | 0 | 1 |  |  | 0 | 0 |  |  | * | = |  |
| *Plagiomnium undulatum* (Hedw.) T.J.Kop. | 4 | 4 | 0 | **33.3** | **0.0556** | 4 | 0 |  |  | 0 | 0 |  |  | * | = |  |
| *Rhytidiadelphus squarrosus* (Hedw.) Warnst. | 2 | 2 | 0 | **33.3** | **0.0532** | 0 | 2 |  |  | 0 | 0 |  |  | * | = |  |
| *Thuidium delicatulum* (Hedw.) Schimp. | 9 | 9 | 0 | **33.3** | **0.0574** | 6 | 3 |  |  | 0 | 0 |  |  | V | ? | V |
| *Trichodon cylindricus* (Hedw.) Schimp. | 1 | 1 | 0 |  |  | 0 | 1 |  |  | 0 | 0 |  |  | * | = | * |
| *Warnstorfia fluitans* (Hedw.) Loeske | 1 | 1 | 0 |  |  | 1 | 0 |  |  | 0 | 0 |  |  | V | ↓ | V |
| **Species occur on both types of meadows** |  | | | | | | | | | | | | | | | |
| *Amblystegium serpens* (Hedw.) Schimp. | 11 | 6 | 5 |  |  | 9 | 2 |  |  | 3 | 2 |  |  | * | = |  |
| *Barbula convoluta* Hedw. | 4 | 2 | 2 | **27.3** | **0.0714** | 2 | 2 |  |  | 1 | 1 |  |  | * | = | * |
| *Brachythecium mildeanum* (Schimp.) Schimp. | 35 | 6 | 29 |  |  | 20 | 15 |  |  | 15 | 14 |  |  | V | ↓ | D |
| *Brachythecium rutabulum* (Hedw.) Schimp. | 54 | 10 | 44 |  |  | 38 | 16 | **24.9** | **0.0018** | **32** | **12** | **36.3** | **0.0076** | * | = |  |
| *Calliergonella cuspidata* (Hedw.) Loeske | 90 | 50 | 40 | **72.3** | **0.0106** | 57 | 33 | **27.9** | **0.05** | 24 | 16 |  |  | * | = |  |
| *Drepanocladus aduncus* (Hedw.) Warnst. | 16 | 10 | 6 |  |  | 12 | 4 |  |  | 2 | 4 |  |  | * | = | D |
| *Fissidens adianthoides* Hedw. | 14 | 10 | 4 | **55.7** | **0.0134** | 12 | 2 | **8.7** | **0.0200** | 3 | 1 |  |  | V | ? | 3 |
| *Oxyrrhynchium hians* (Hedw.) Loeske | 57 | 23 | 34 |  |  | 25 | 32 |  |  | **7** | **27** | **46.6** | **0.0002** | * | = |  |
| *Plagiomnium affine* (Blandow ex Funck) T.J.Kop. | 19 | 14 | 5 | **73.7** | **0.0008** | 10 | 9 |  |  | **0** | **5** | **10.4** | **0.0150** | * | = |  |
| *Pseudoscleropodium purum* (Hedw.) M.Fleisch. | 28 | 24 | 4 | **77.7** | **0.0008** | 21 | 7 |  |  | 1 | 3 |  |  | * | = |  |
| *Sciuro-hypnum oedipodium* (Mitt.) Ignatov & Huttunen | 8 | 5 | 3 | **40.9** | **0.0574** | 6 | 2 |  |  | 3 | 0 |  |  | * | = | V |
| **Species occur only on restoration sites** |  | | | | | | | | | | | | | | | |
| *Brachythecium albicans* (Hedw.) Schimp. | 1 | 0 | 1 |  |  | 1 | 0 |  |  | 1 | 0 |  |  | * | = |  |
| *Brachythecium rivulare* Schimp. | 2 | 0 | 2 |  |  | 2 | 0 |  |  | 2 | 0 |  |  | * | = | * |
| *Hygroamblystegium varium* (Hedw.) Mönk | 1 | 0 | 1 |  |  | 0 | 1 |  |  | 0 | 1 |  |  | * | = | D |
